# President’s Address to the American Dental Association, 1897

**Published:** 1897-09

**Authors:** James Truman


### PRESIDENT'S ADDRESS TO THE AMERICAN DENTAL ASSOCIATION, 1897.

BY JAMES TRUMAN, D.D.S.

The annual gathering of a body such as this constitutes an ever-recurring era in dentistry, and should mean, to the reflective mind, a period of advance along all lines of work. If this fails to be realized, then the year has been valueless, and should be regarded as a period lost in the progress of the race.

The interest that has been manifested everywhere since the close of the last session of the American Dental Association, at Saratoga, indicates that the dental profession is fully alive to the importance of this meeting at Old Point Comfort, and of the possibilities which may accrue from its deliberations. I, therefore, wish to congratulate you on the fact that those here assembled have met upon a mission not second in importance to any of the meetings that have preceded this; and it may, perhaps, when the results have all been summed up, transcend all others; for, if the feeling be judged aright, we have reached the "parting of the ways," and the rising sun of another day already appears above the horizon, illuminating the approaching century with a golden halo, indicating brilliant but as yet unfathomable possibilities.

The work of the past year will compare favorably with that of those preceding it, yet it must be conceded that the spirit of activity is not abroad in the land or in our profession. It is unfortunately too true that our calling tends to physical depression and disinclination to that higher work so necessary for advancement. The physical slavery which practice entails is not conducive to active mental effort, and the exhaustive day inclines to rest rather than to renewed activity during the evening hours. The result of this enforced inertia is to self-sacrifice, and the man of investigating mind will turn indifferently from the bank account to the solving of problems, and eventually he becomes not a practitioner but a laboratory devotee. Through this division of labor there has arisen a very small class who are the prophets of the age, and these are they to whom we have learned to look for the new ideas which mark progress.

The various reports will call to your attention the things new and old of special interest. It will suffice here to say that the practical side of dentistry has not exhibited any features during the year worthy of special comment. This, however, must be said, that the literature of this portion of our work has been greatly enriched by several notable productions, and while there has been little absolutely new, there has been marked improvements upon older methods of work.

The uncertainties attending the introduction of cataphoresis has received careful practical observation and quite thorough experimental tests, and this process is gradually finding its true level in practice. Its adoption by the dental profession has been certainly phenomenal, and this constitutes its greatest danger, for the unwise rush in and attempt the impossible, forgetting that pain is the sentinel upon the advanced picket-line and an ever-present warning of danger. To use this method to the best good of the patient means a high order of anatomical knowledge, as far as the teeth are concerned, and this point needs more and more to be impressed upon the dental mind everywhere.

The work in what may be called the scientific side of dentistry has received fresh impulse through the labors of Black, Andrews, and Williams, and the labors of dentists in England and on the continent have advanced our knowledge and aided in the solution of many abstruse problems. That a large portion of this work has been accomplished by Americans is worthy of special mention at this time. It is recognized, however, that science knows no nationality, and any attempt to erect boundary lines should be crushed in its inception; but, while this is true, the fact remains, and it is one worthy of self-congratulation, that at last in our history a class has arisen in this country the equal of any similar body of unselfish men to be found among the older civilizations and worthy of the highest honor. This class must ever remain a small one, but it is to its efforts that we must look for the ennobling of our race on this continent in all the higher walks of intellectual life. In this connection it is befitting the occasion that we express our deep regrets and sympathy that the most brilliant exponent of this class, Professor W. D. Miller, is physically suffering from the great and exhaustive labors to which he has subjected himself. It is felt that all here assembled will send back to him the expressed desire that he may know a full restoration to health and activity.

The year has brought with it serious losses in our ranks, both at home and abroad. It is proper that we should stop in the busy whirl of daily duties and consider what these men were to us and what we owe to their memories. Their mission on earth has been completed, but the work they did is the precious heritage of their profession, and it should be our duty to treasure it, and add thereto, that the incoming century may feel the diviner inspiration to more exalted ideals.

The Committee on Necrology will, doubtless, present suitable resolutions upon the character and work of our revered members who have passed from the shadow of things to the eternal verities of the life everlasting.

To review a year is not difficult, but to grasp the progress made in a century is a problem from which the wisest and most cultivated might well shrink in dismay. The attempt will not be made here, but the near approach to the close of the nineteenth era of Christian brotherhood naturally causes a retrospection, and the query is ever uppermost, What have we stored up for our profession in the past one hundred years? It is a very glib expression, upon the tongue of the unthinking, that all we know of dentistry worthy the name has been acquired in this century. While the period has been fruitful in progress, the claim can never be made that we owe little or nothing to the one hundred years preceding. The intelligent student of dental history soon comes to recognize that we must moderate our claims and give credit to the last century as being worthy of our highest regard. It must be learned, further, that this child of the professions—dentistry—was not born in America, but was nurtured in France, England, and Germany, and developed there upon a broad experience that made its fractional life here in America but a continuation of that of the years preceding.

While this is gratefully conceded, it must be acknowledged that dentistry has shown an increased vitality upon this continent, and the progress made has been proportionately greater here than elsewhere. Neither in the Old World nor in the New was there any real advance in this work until near the close of the last century, when porcelain teeth were introduced by Chemant, of France. Then, in the present century, came the introduction of arsenic for the devitalization of the pulp by Spooner, of Montreal; the filling of the canals; the revolution in surgery by the discovery of anæsthesia by Wells, and the introduction of ether by Morton, both dentists; the introduction of cohesive gold by Arthur; the adoption of the mallet, and the invention of the electrical mallet by Bonwill; the invention of the various forms of dental engines; the rubber dam, and, finally, the use of electricity as a motive power and as an obtundent. These are the prominent and marked stages which have been taken, each aiding to carry us forward to our present position. The countless details of minor improvements cannot even be mentioned here, but without their aid, all present know that the practice of dentistry would, to many, be almost impossible.

The crowning glory of the century, next to that of the discovery of anæsthesia, has been the solution of the problem of the ages,—caries of the teeth. While giving due prominence to the labors of a host of active investigators from the days of Leeuwenhoek down through Erdl, Ficinus, Klencke, Leber and Rottenstein, Milles and Underwood, Magitot, Tomes, we must give the credit for the final completion of the great work to Professor W. D. Miller, resident of Berlin, Germany, but native of this country. It is a special cause of congratulation that this investigation has been completed in this era and during the two decades that close the century.

It is also worthy of historical mention that dental education received its first impulse in Baltimore, Md., the adjoining State to that in which we are now assembled, Virginia, the Mother of Presidents. The wave thus started has been widening its circles ever since until it has embraced all civilizations. The progress in this direction is unexampled in professional educational work, and it is safe to assume that it has not only reached an equality with that of general medicine, but, in some respects, has led that venerable mother into paths she had not thought of treading. The steady advance in the standard of dental education, the extension of the curriculum, the erection of noble buildings for dental training, the grasp of new ideas, are all signs of that activity which makes for progress. It was a natural sequence to this when Professor Winder, dean of the Original School of Dentistry, suggested the formation of a legislative body for all dental colleges, and the outgrowth of this idea was the formation of the National Association of Dental Faculties, not the least of the remarkable products of the century. To this organization we owe all of the advances made in dental education in the United States, and we, as a profession, may look forward with trustful confidence that its work in the future will maintain the high record it has earned in the past.

The natural and final result of advanced thinking and acting is law. A world without law would represent chaos, if such a condition be thinkable. Law, in the professional sense, is the embodiment of orderly rules of conduct, and should be recognized as necessary for the good government of dentistry as well as all organized bodies. The result of educational advance meant a proportionate demand for restriction,—law making. There is the natural law of all things which works for harmony, and there is the man-made law, which is very frequently a source of inharmony. The final outcome of this legal tendency has been laws in every State, a National Association of Dental Examiners, and a general feeling of disquiet among all teachers of dentistry as a consequence. Whether this product of the nineteenth century is to result in the good anticipated by those who worked for it remains yet to be seen, but it is hoped that this Association will see the way clear to formulate something that will tend to quiet the growing antagonism engendered by the unwise multiplication of statutes. The subject is a large one, and has frequently claimed the attention of my predecessors in office. As these laws at present stand, they are a dangerous obstacle in the path of professional progress, and promise to be the one blot upon the otherwise fair fame of the century in dental work. This applies not specially to individuals, for it is recognized that it is mainly the outgrowth of a mistaken sentiment that force, through law, will accomplish everything. The aim should be for unity of effort with the least friction State with State, accompanied with a positive recognition that the decree of one State should, in this matter, be a law for every citizen of the

United States. The Constitution of the United States expressly declares, Article IV., Section 1, "Full faith and credit shall be given in each State to the public acts, records, and judicial proceedings of every other State, and the Congress may by general laws prescribe the manner in which such acts, records, and proceedings shall be proved, and the effect thereof.

Section 2. "The citizens of each State shall be entitled to all privileges and immunities of citizens in the several States."

These quotations are apparently clear, and unmistakably point to two facts, that full faith and credit shall be given in each State to all the acts of every other State, and that Congress shall prescribe the effect of all such laws and how such shall be proved; and, further, that a citizen of one State shall be entitled to all the privileges of citizens of the several States. It is, therefore, clear that a law passed depriving a citizen who has been declared legally entitled to practise in any State from the privilege of registration in another State is unconstitutional, and it is difficult to understand how the Supreme Court of the United States could decide otherwise if a case should be carried before it for adjudication.

Out of forty-three States and Territories having laws regulating the practice of dentistry, in thirty-two the Examining Boards are appointed by the governors. In eight the State societies have the selection. In one the Board of Public Works selects. In another the selection of the board is partially made by the governor. In another the appointments are made by the Board of Health, and in the District of Columbia by the Commissioners of the District.

The graduates of the dental colleges of this country are subjected to the final decision of men who are not accountable to the dental profession, but owe their places to political preference. The boards may be, and doubtless are in many cases, composed of men worthy to fill such responsible positions, but how is it possible to determine this fact? It is not surprising, in view of the origin of the large majority of these boards, that the National Association of Dental Examiners should attempt to declare what should or should not be the curriculum of each of the dental colleges of the United States. The only apology that can be offered for this insidious blow at dental education is that it emanated from men who have no familiarity with dental teaching, and who have based their conclusions on impracticable theories.

It is not possible, perhaps, for the American Dental Association to legislate to correct this growing evil of State boards, as such action would be inoperative. It can, however, use its powerful influence, as the representative body of American dentists, to have all places on the boards filled upon the recommendation of State dental societies or by bodies having similar power. The suggestion made that persons to be eligible for State examiners should themselves be examined is certainly worthy of consideration, but at present there is no power to compel this, and it will be a long time, in the future, before a general law by Congress will be passed covering professional education in the States.

The deep responsibility of this occasion must be felt by every one here assembled. It means the old interrogatory, old as humanity, What shall we do to effect our salvation as a national organization? The hour has come for action, but the hour brings with it thoughts new and old. The past historical record of the American Dental Association is replete with undying memories, and the work accomplished should stir the mind to solemn reflections. In the background stand our dead brothers, and we can most truly believe that they point with uplifted arms to the future, and from the unseen comes the command, March on! For the progress of professions, as well as civilization, can only be accomplished through changes, the death of the old, the installation of the new.

The organization of societies in dentistry in the United States is but little over a half-century old. The condition of things in the dental profession in the thirties can scarcely be conceived by the liberal minds of the present. It was a period of isolation. The hand of every man was against his fellow-practitioner. The walls of prejudice were erected everywhere. Doors were bolted and barred against intrusion. Lips were sealed, lest they might disclose an important method of practice. Nowhere, throughout this progressive land in dentistry, was there any light upon the mediæval darkness. It, however, proved to be the period before the dawn. From behind all the clouds and shadows there was heard a voice proclaiming in no uncertain tones the old cry, "To your tents, O Israel!" but it fell upon deaf ears. It was the voice of Horace H. Hayden. With true prophetic inspiration he ceased not to demand that the dentists of the time should come forth from their isolation and, with something of the spirit of the old crusaders, unite and arm for the approaching conflict. The day came when Horace H. Hayden conquered, and in New York the first society was born into life, but died in its birth. Again we find this courageous pioneer at the head of the American Society of Dental Surgeons, and then, for the first time on this continent, began the true life of associative dental effort. We rear monuments to our great men and women, but the only monument yet reared to Horace H. Hayden lies in the historical record of his deeds. Upon his neglected tomb I would lay this chaplet that the dental profession may never forget what it owes to this one man who labored that we to-day may live.

The past of the American Dental Association is familiar to all. It is a worthy record. It began its life amidst much confusion as to organization. It was the conservative embodiment of that intermediate thought between the crushing power sought to be enforced by the old American Society of Dental Surgeons and that extreme liberal sentiment embodied in the American Dental Convention. The organizers of the American Dental Association builded well for their day and generation, with the result that it has continued its active life for thirty-eight years. Has it fulfilled its destiny? Time alone can answer this profound question. We are here to-day to meet with our Southern brethren to attempt the solution of a difficult problem. The affectionate interest of long association lingers around their organization as well as our own. Is the ultimate result to be that we meet to-day, on consecrated ground, to bury our past loves, to scatter the elements of separation to the four winds of heaven, and over the ashes erect the new temple of which the fathers had no conception, a union of all professional thought upon a foundation that will sustain the superstructure for many years, it is hoped, far into the coming century?

The conditions under which the American Dental Association was organized made it impossible to effect radical changes in old methods. The period was replete with crudities in practice and theory. Colleges were few and not well arranged for the training of students. Ignorance prevailed of those scientific studies which were rightly regarded as essential to a true knowledge of the human organism. It was, therefore, impossible to form a strictly scientific body. Is this the condition at present?

Year by year the standard of training has been increased in our colleges through the wise action of the National Association of Dental Faculties, bringing us to a period of enlarged culture, enabling us to view calmly, and consider well, our relations to the present and to the future.

The organization of a body such as has been contemplated is a serious matter, and should not be hastily entered upon. It would not be proper in this address to do more than suggest a path that would seem to be the proper one to follow.

We live in the present, but must build for the future, or that future will take the matter out of our hands and destroy the work of this day and generation. It is feared that the importance of this is not fully appreciated. The basis of all dental national organizations is derived from the American Medical Association. This, as stated, served its purpose well for that body, but in the American Dental Association has tended to weakness, so much so that the cry has gone over the land, the American Dental Association has ceased to be a body worthy our support! While this at no time has been true, the fact that such a statement has been heralded everywhere is indicative of a feeling that old methods have subserved their purpose, and a new order of things is demanded. In view of this, it would seem as though reorganization must, to be effective, be based upon an entire change of methods. Without attempting a solution of this matter, it may serve a purpose to point out what may be regarded as some things worthy of change in the old Association. The first step in a national professional organization should be the proper arrangement of local societies. From these the national body must be formed. These local societies must be in direct connection with the National, and therefore be established upon a careful, ethical basis, and a positive standard of entrance. This, as a rule, is entirely neglected, resulting in a membership of uncertain quality. The representation to the national body is selected with more regard to availability than to qualifications. These delegates, if so disposed, became permanent members of the national organization, and eventually became part of the controlling body. In proportion as this grows will it become unmanageable and lose its scientific character. This is the history of all similar bodies so constituted. Science does not, cannot flourish in crowds.

The question, then, would seem to be, Can reorganization be accomplished upon a different conception? It will be difficult to change present methods in local bodies, but proper legislation in the national organization could in time effect much. A glance at the present and past condition of the American Dental Association may prove of value. The ineffectual efforts to increase the scientific work led to the formation of sections, under the supposition that these would tend to concentrate the labor and accomplish more than when left to the efforts of committees. The result has been no improvement over older systems. This is not stated as a complaint, for it is recognized not to be the fault of individuals but of methods. The system is intrinsically weak, and can never effect any permanent good results.

The first fatal injury done to any organization is to found within its borders an aristocracy. This has been accomplished in this and similar organizations by the establishment of what is known as permanent membership. This has been the deadly poison that has eaten out the life of this American Dental Association. It was an aristocracy not based, as it should have been, as a prize for higher work, but was upon ability to pay the yearly fee and attendance at the annual meetings. This separated most of the desirable element in the profession, and, as a consequence, very little of the real scientific work has been done within its borders.

If these views be accepted, it would seem worthy of our careful thought whether it is not time to originate a body distinct from all others, and which should embody elements of strength rather than weakness.

The idea which this address seeks to impress is that a national body should be the culmination of all below it. It should embrace the concentrated wisdom of all subordinate societies. In order to make it thus the affiliating organizations should send to the national only such members who had given the profession satisfactory work. Their entrance and continuation in the national could be arranged so that the latter would always be an active, living organization. The only aristocracy admissible should be a class, set apart, who had earned special honor. This would never be a large number, and might be selected from the main body with an honorary title.

With this care in the selection of members the national body would always remain within working limits, and would partake of the character of the higher scientific bodies of older civilizations.

The suggestions here outlined will not in all probability meet the views of the majority, but it is hoped that if a new body be formed out of the two present organizations that it will embody, at least, some improvements over those long in existence. They have been tried and found wanting in those essentials that work for the highest good of the dental profession. It will not be disputed, it is presumed, that a national body is a necessary part of the organization of a profession. This recognized, it should be the aim of those having the matter in charge to make it worthy the respect not only of the present time but of the future.

The recent meeting of the American Medical Association in Philadelphia demonstrated that the divisions into sections, to meet separately from the main body, is not a satisfactory method. It is questionable whether much good is derived, even in medicine, from this practice, where specialties are more clearly pronounced and certainly more desirable; but in dentistry there is no such demand. We are, or should be, interested in every portion of our work, and can, therefore, meet on common ground, enlarging our horizon of thought and experience, and at the same time cultivate those social amenities so important in maintaining good fellowship among widely separated members of the same professional family.

The experience of the past few years has demonstrated that it cannot be expected that the regular practitioners of dentistry will, outside of a few isolated instances, work for the advance of his profession, if it interferes with his daily work. The altruistic side of dentistry needs cultivation, but it will never assume large proportions. The true investigator of problems invariably finds practice irksome, and he is always ready to burst the bonds, if necessary, which environ him for the more enjoyable fields of the mysterious unknown. Few there are who can do this with that other problem—daily bread—staring them constantly in the face. It would seem, therefore, that the time has arrived when some means should be arranged to set free these imprisoned students and make it possible for them to work independently of the cares of life. When the new organization is perfected it is hoped the possibilities, in this direction, may be considered, and that a fund may be established looking to financial aid to those who are continually sacrificing themselves, and those depending on them, that the profession may progress worthy of its high aspirations. Science seeks the cloisters in the world's life. Is it not time that we make those cloisters free from worldly anxieties?

With our faces averted from the past and present, and directed to the opening of the new day, we can consistently ask, What of that great future that lies before us? Who has the prescient wisdom to fathom its possibilities? It is the as yet uncultivated plain; its mines have not been laid bare with pick and shovel; its gold and precious stones still remain environed by the accumulated mire and rocks of undetermined time; but over all these floats the spirit of progress, and her wings are never idle, nor will she be found wanting in inspirational power to influence men to work out the problems of the coming ages. The promises of that future will assuredly meet with full fruition, and we can confidently anticipate that, inasmuch as we have builded well with imperfect tools, they who come after us will rear more substantial structures and carry the profession of dentistry to a standard as yet unthought of in our wildest imaginings.

Men of the North, South, East, and West, here assembled, on ground consecrated by the martyrdom of many heroic spirits, can you not come together actuated by that same high sense of duty that led the thousands to sacrifice all of life's treasures for the good of others, and pledge yourselves to a renewal of effort to complete brotherly relations, and, forgetting the old and grasping the new, move onward in fraternal harmony towards the anticipations, the possibilities that lie entombed in the ever-developing future, the heritage of the incoming civilizations, which should prove the blessing of humanity; the ennobling of the professions, and the upbuilding of the life and intelligence of all the peoples of the earth.

